# The association between food insecurity and poor sleep quality among transgender women of color: The TURNNT cohort study

**DOI:** 10.1080/30678935.2026.2713222

**Published:** 2026-09-28

**Authors:** Alexander Furuya, Nour Makarem, Adam Whalen, Jenesis Merriman, Denton Callander, Asa Radix, Miho Kawanami, Dustin T. Duncan

**Affiliations:** a Department of Epidemiology, Columbia University Mailman School of Public Health, New York, New York, USA; b Kirby Institute, University of New South Wales, Sydney, Australia; c Department of Epidemiology, Callen-Lorde Community Health Center, New York, New York, USA

**Keywords:** Transgender health, food insecurity, sleep health, social determinants of health, social epidemiology

## Abstract

**Introduction:**

The purpose of this study is to quantify the longitudinal association between food insecurity and sleep health among transgender women of color (TWOC).

**Methods:**

We used longitudinal survey data from 314 TWOC (2020 to 2023). Our sleep variables were short sleep (sleeping for less than seven hours), poor sleep quality (self-rated sleep quality as “Very” or “Fairly bad”), and long sleep onset latency (experiencing sleep onset latency at least three times a week). We conducted bivariate analyses to identify correlates of food insecurity. We used Targeted Maximum Likelihood Estimation to estimate the adjusted risk ratios, adjusting for age, education, income, US-born nativity, sex work history, and incarceration history.

**Results:**

Among the 314 participants, 59.9% reported experiencing food insecurity, 54.5% had short sleep, 35.0% had poor sleep quality, and 25.2% experienced long sleep onset latency. Those with lower income and less education were more likely to experience food insecurity. We found that food insecurity measured at baseline was associated with poor sleep quality measured at six-month follow-up (Adjusted Risk Ratio: 2.03; 95% Confidence Interval: 1.26, 3.29).

**Conclusion:**

These findings underscore the need for sleep interventions that address the structural determinants of food insecurity among TWOC.

## Introduction

Transgender women of color experience high rates of food insecurity, defined as the economic and social conditions that limit access to adequate food (Bleich et al., 2023). The prevalence of food insecurity among transgender women of color is estimated to be 44.0%; in the general population, it is estimated to be around 13.5% (Rabbitt et al., 2025; Zubizarreta et al., 2024). These elevated rates imply that a large number of transgender individuals are potentially experiencing hunger and undernutrition every day (Ayala & Meier, 2017). In addition to experiencing hunger, those experiencing food insecurity are more likely to have physical health issues, including cardiovascular disease, diabetes, and certain types of cancers (Odoms-Young et al., 2024), as well as mental disorders, including depression and anxiety (Ejiohuo et al., 2024).

According to intersectionality theory, transgender women of color are vulnerable to food insecurity because of the unique forms of structural oppression that affect nearly every aspect of their life, including employment, housing, and health care (Crenshaw, 1991; Smart et al., 2022; Wesp et al., 2019). Racism, transphobia, sexism, and classism can hinder food security in many ways for transgender women of color. For example, systems of oppression can put them at a socioeconomic disadvantage and poverty, limiting their access to fresh and healthy foods (Odoms-Young et al., 2024). Additionally, historically racist policies such as redlining physically place transgender women of color in residentially segregated areas that are lacking in supermarkets and grocery stores, but abundant in fast-food restaurants (Bowen et al., 2021; Leslie et al., 2022). Food insecurity, therefore, should not be thought of as an individual problem, but rather as a systemic issue (Swales et al., 2020).

Transgender women of color are also at an increased risk of adverse sleep health (Caceres et al., 2019; Duncan et al., 2021). We previously found that 54.8% of transgender women of color reported having shorter sleep duration (i.e., less than 7 h per night) (Duncan et al., 2021); this rate is higher than that of the general population in the US (33.2%) (Pankowska et al., 2023). Furthermore, we previously found that 29.0% of transgender women of color viewed their sleep quality as poor (i.e., “fairly bad” or “very bad”) (Duncan et al., 2021); this is comparable to that of the general population in the US (Knutson et al., 2017). Those experiencing shorter sleep and worse quality sleep are more likely to have obesity, anxiety symptoms, depression, and practice risky health behaviors (Colten & Altevogt, 2006). Despite extant inequalities in sleep factors by sexual orientation and gender identity (Patterson & Potter, 2019), sleep remains understudied among sexual and gender minority populations (Duncan et al., 2021, 2022; Leonard et al., 2025). The limited existing research on sleep among transgender populations demonstrates that sleep health is a determinant of physical and mental health for the population; for example, a study of transgender women and transgender men in Germany identified poor sleep quality as the strongest predictor of poor quality of life (Auer et al., 2017).

In the general population, there is an association between food insecurity and sleep health (Alhasan et al., 2023). According to a systematic review and meta-analysis, among the general population, food insecurity increased the risk of poor sleep quality (Odds Ratio: 1.45; 95% Confidence Interval: 1.24, 1.70) (Mazloomi et al., 2023). There are plausible mechanisms between food insecurity and sleep health. Food insecurity may cause adverse sleep health in a mechanism involving stress and psychological distress; some researchers hypothesize that food insecurity causes stress, which can then lead to sleep disturbances.(Arzhang et al., 2024; Bermúdez-Millán et al., 2016) Additionally, those experiencing food insecurity may be more likely to consume convenience foods high in sugar and fats and low in nutrients, which can lead to worse sleep health (Ding et al., 2015; Mazloomi et al., 2023)

Yet, little is known about the association between food insecurity and sleep health among sexual and gender minority individuals. Transgender women of color may experience food insecurity and sleep health in ways different from those of the general population, given the unique forms of discrimination they may encounter and their unique lived experiences (Smart et al., 2022). Thus, assuming that the results from the general population would apply to transgender women of color may be incorrect. Using pilot baseline data from a cohort of transgender women of color, we found an initial association between sleep duration and food insecurity (Duncan et al., 2021). However, because the research design was cross-sectional in nature, we could not discern whether food insecurity caused adverse sleep health, or if adverse sleep health caused food insecurity; additionally, the pilot sample included only 31 women, limiting statistical power and the ability to do complex analysis.

The overall objective of this study is to quantify the longitudinal association of food insecurity and sleep health among transgender women of color. Understanding determinants of sleep health can illuminate potential interventions that can reduce health inequities. Furthermore, understanding the association between food insecurity and sleep health can help us understand sources of health inequalities that transgender women of color experience. There are several aims of this study: first, to quantify the prevalence of food insecurity and sleep health outcomes among transgender women of color; second, to identify potential correlates of food insecurity; and third, to characterize the association between food insecurity and sleep health among transgender women of color. We take advantage of the longitudinal data from Trying to Understand Neighborhoods and Networks Among Transgender Women of Color (TURNNT) Cohort Study to address these research aims.

## Materials and methods

### The TURNNT cohort study

We used longitudinal data from 314 transgender women of color from the TURNNT cohort study, where we recruited participants using convenience sampling methods, including social media marketing, event-based recruitment, referrals from organizational outreach, and snowball sampling *via* referrals from enrolled participants (Callander et al., 2020; Duncan et al., 2024). The research team recruited participants from August 31, 2020, to November 4, 2022; each participant was followed up for a year, and data collection was completed on December 19, 2023. Data collection for each participant occurred at three distinct waves: baseline, 6-month follow-up, and 12-month follow-up. Participants were compensated with pre-paid gift cards as follows: $50 per interview at enrollment, $75 per interview at the 6-month follow-up, and $100 per interview at the 12-month follow-up.

Eligible participants identified as transgender women of color, were between 18 and 55 years old at enrollment, and lived in the New York City metropolitan area (Furuya et al., 2025). To determine eligibility, we asked potential participants what race and gender they identified with. For race, we asked: “There are many different ways that people choose to describe their ethnicity and race. From the list that I read, please indicate those that are relevant to you, noting that you may choose more than one option. Do you identify as ‘Black/African American’, ‘Latina/Latino/Hispanic’, ‘Middle Eastern/North African’, ‘Native Hawaiian/Pacific Islander’, ‘White/European American’, ‘American Indian’, ‘Asian/Asian American’, ‘Biracial/Multiracial’, or ‘Something Else’?” Participants who selected exclusively “White/European American” were excluded from the study. Participants reported their gender and assigned sex at birth at the beginning of the survey. For those who indicated identifying as a transgender woman, we confirmed their identity as a transgender woman with the following question: “Do you identify as either a woman, a transgender woman, or a woman with transgender experience?” Those who confirmed yes to this question were included in the TURNNT study.

The TURNNT cohort and study design were developed in close collaboration with community leaders from the transgender women of color community and service organizations that support their health (Duncan et al., 2024).

### Ethics statement

All procedures performed in studies involving human participants were in accordance with the ethical standards of the institutional and/or national research committee and with the 1964 Helsinki declaration and its later amendments or comparable ethical standards. The study was approved by the Institutional Review Board at Columbia University Irving Medical Center (IRB-AAAS8164).

At the initial enrollment interview, we provided interested individuals a consent form describing the study background, requirements, timeline, confidentiality procedures, and participant rights. Participants gave consent voluntarily by agreeing to the terms of the study and signing the consent form. Informed consent was obtained from all individual participants included in the study. Informed consent for publication was provided by the participants when they consented to participate in the study.

### Food insecurity

We measured baseline food insecurity using a single-item question adapted from the American Academy of Family Physicians’ Social Needs Screening Tool (O’gurek & Henke, 2018). We asked participants, “Within the past 6 months, were you ever worried whether your food would run out before you got money to buy more?” Participants could respond “Yes,” “No,” or “Don’t know.” We coded “Don’t know” as missing and examined this variable as a dichotomous variable. We measured food insecurity at every wave.

### Sleep health

The original Pittsburgh Sleep Quality Index (PSQI) includes 19 items to measure the construct of sleep quality and disturbances (Buysse et al., 1989). For the TURNNT study, we used a subset of items from the PSQI to capture different aspects of sleep health. In the present paper, we used measurements of sleep duration, sleep quality, and sleep onset latency for our outcome of interest. We measured sleep duration and sleep quality at baseline, six-month follow-up, and twelve-month follow-up; we only measured sleep onset latency at six-month follow-up and twelve-month follow-up. For sleep duration, we asked participants to report a numerical value for how many hours of sleep they believe they get at night. We characterized those who reported sleeping for less than seven hours as getting insufficient sleep; the seven-hour cutoff has been used by other sleep health researchers and has clinical significance for other health outcomes (Watson et al., 2015; Whalen et al., 2025; Chaput et al., 2020). For sleep quality, participants rated their subjective sleep quality as “Very good,” “Fairly good,” “Fairly bad,” and “Very bad.” We operationalized this sleep quality as a dichotomous variable. Those who responded, “Very good” and “Fairly good” were classified as having good sleep quality, whereas those who responded, “Fairly bad” and “Very bad” were classified as having poor sleep quality. Finally, for sleep onset latency, we asked participants: “During the past month, how often have you not been able to fall asleep within 30 min?” Those who reported taking longer than 30 min to fall asleep three or more times a week in the past month were classified as having long sleep onset latency; this cutoff is the most similar to the International Classification of Sleep Disorders definition of insomnia disorder (Markwald et al., 2018).

### Potential confounders

Based on the extant literature, we believe there are several confounders that may be common causes of both food insecurity and sleep health, including age, socioeconomic status (i.e., education and income), and factors reflecting one’s lived experience (i.e., US-nativity, sex work history, and incarceration history) (Atoloye et al., 2026; Barreto et al., 2019; Harrington Meyer & Heflin, 2026; Philippens et al., 2022; Testa et al., 2025; Walker et al., 2021). Therefore, we measured sociodemographic characteristics that may be potential confounders at the baseline survey. We collected data on baseline age (categorized as 18–24 years, 25–34 years, 35–44 years, and 45–55 years), education (less than high school, high school or equivalent, more than high school), individual income (no income, $1 to $9,999, $10,000 to $29,999, $30,000 or greater, or missing as an informative category), US-nativity (US-born or foreign-born), sex work history (ever engaged in or never engaged in sex work), and incarceration history (ever been incarcerated or never been incarcerated).

### Statistical analysis

First, we conducted descriptive analyses to characterize the sociodemographic composition of the study sample as well as to estimate the distribution of food insecurity and measures of sleep health. Second, to assess whether loss to follow-up resulted in changes in the study population’s composition over time, we calculated descriptive statistics for each wave of data collection (baseline, six-month, and twelve-month) and conducted a chi-square test to see if frequency counts changed over time. Third, in order to identify potential correlates of food insecurity, we estimated the cross-sectional bivariate associations (prevalence ratios [PRs]) between sociodemographic characteristics and food insecurity.

Finally, to estimate the adjusted longitudinal effect of food insecurity on sleep outcomes, we used a doubly robust method, Targeted Maximum Likelihood Estimation (TMLE). Using doubly robust approaches can protect against bias due to model misspecification (Li & Shen, 2020). Furthermore, TMLE supports theory-driven machine learning approaches and can therefore incorporate ensemble algorithms such as SuperLearner to further reduce bias arising from model misspecification (Luque-Fernandez et al., 2018). To establish temporality, we used sleep health measured at the six-month and twelve-month follow-ups. To conduct TMLE analysis, we used the LTMLE package in R (Lendle et al., 2017). We adjusted for theoretical confounders, including age, education, income, US-nativity, sex work history, and incarceration history; we adjusted for these variables because we believe they adequately control for confounding while maintaining model parsimony, helping us avoid unnecessary complexity or multicollinearity. Given that there were low rates of missing data, we opted to use a complete case analysis.

All quantitative analyses were conducted using R/RStudio (Version 2026.01.1 + 403), and all statistical tests were conducted at a significance level of 0.05.

## Results

In total, 314 participants who identified as transgender women of color were included in this analysis. We calculated descriptive statistics for the baseline characteristics of the study population (Table 1). There were 218 (69.4%) participants who completed the 6-month follow-up and 193 (61.5%) who completed the 12-month follow-up, resulting in a smaller analytical sample over time. At baseline, 59.9% of the participants reported experiencing food insecurity; this largely did not change by the 6-month follow-up (57.3%) or 12-month follow-up (59.6%). In terms of sleep duration, more than half (54.5%) had inadequate sleep at baseline; at 6-month follow-up, the prevalence was 52.8% at 6-month follow-up, and at 12-month follow-up, it was 56.5%. For sleep quality, 35.0% had poor quality sleep at baseline; this decreased to 29.3% by 6-month follow-up, then to 28.5% by 12-month follow-up. At 6-month follow-up, 25.2% had experienced long sleep onset latency, and this changed to 28.0% by 12-month follow-up. When we examined characteristics of the study population over time, we found that none of the covariates changed significantly (Supplemental Table 1); the rate of missing data for any given variable stayed consistent over time.

From our cross-sectional bivariate analysis, we identified several correlates of food insecurity (Table 2). Compared to those who reported an annual income of $30,000 or greater, participants who reported an income between $10,000 and $29,999 were 87% more likely to report food insecurity (PR: 1.87; 95% CI: 1.17, 2.99); this was similar for those who reported an income between $1 and $9,999 (PR: 1.71; 95% CI: 1.08, 2.71) and those who reported no income (PR: 2.12; 95% CI: 1.33, 3.38). Education was a significant predictor, too; those with less than a high school education were 28% more likely to report food insecurity compared to those with more than a high school education (PR: 1.28; 95% CI: 1.04, 1.59). We did not find any statistically significant associations between food insecurity and age, US-born nativity, sex work history, or incarceration history.

Here, we present the adjusted associations, which can be found in Table 3. We found that there was an association between food insecurity measured at baseline and poor sleep quality measured at six-month follow-up, even after adjusting for age, education, income, US-born nativity, sex work history, and incarceration history (Adjusted Risk Ratio: 2.03; 95% CI: 1.26, 3.29). We did not find a statistically significant association between food insecurity and sleep duration or between food insecurity and sleep onset latency. We report the unadjusted associations between food insecurity and sleep outcomes in Supplemental Table 2.

## Discussion

The ways in which food insecurity and sleep affect transgender women of color are not well-documented in scientific literature. Findings from the general population may not be transportable to transgender women of color, given their unique lived experiences; food insecurity and its association with sleep health may operate differently among those with intersecting marginalized identities (Wesp et al., 2019). For example, transgender women of color experience are often disproportionately targeted by law enforcement, which could affect the association between food insecurity and sleep (Furuya et al., 2026). Therefore, this study aimed to address the gaps in the literature by estimating the prevalence of food insecurity, identifying correlates of food insecurity, and quantifying the longitudinal association between food insecurity and sleep health among transgender women of color. Based on our results, we found that: 1) transgender women of color experience high rates of food insecurity, 2) socioeconomic status is associated with food insecurity, and 3) food insecurity is associated with worse sleep health over time.

Our data shows that six in ten transgender women of color in our sample faced food insecurity at baseline, highlighting a disturbing reality among this population. Indeed, these findings confirm that transgender women of color are more likely to experience food insecurity compared to that of the general population (13.5%) (Zubizarreta et al., 2024; Rabbitt et al., 2025). We found that measures of socioeconomic status, namely less than a high school education and income less than $30,000, were associated with food insecurity. We interpret these results to suggest that food insecurity is a result of social deprivation, which is caused by systems of oppression, including racism, transphobia, and sexism (Bowen et al., 2021). Bowen et al. posit that the many facets of racism (including, but not limited to, socioeconomic deprivation, neighborhood racial segregation, mass incarceration, and mass deportation) act as fundamental root causes of food insecurity (Bowen et al., 2021). We concur with this finding and add to that that systems of oppression related to transphobia and sexism also affect transgender women of color.

In terms of sleep, we found that transgender women of color had a higher rate of inadequate sleep (54.5%) compared to that of the general population (33.2%) (Pankowska et al., 2023). The rate of inadequate sleep that we found in our data is similar to that found in the few other studies examining sleep among transgender individuals (Caceres et al., 2022; Leonard et al., 2025). Our study provides the first longitudinal evidence that food insecurity is associated with poorer sleep quality over time among TWOC. Other researchers have found similar findings in different populations (Arzhang et al., 2024; Gyasi et al., 2022).

While other researchers found that food insecurity was predictive of shorter sleep (Duncan et al., 2021; Mazloomi et al., 2023; Troxel et al., 2020), we did not observe this association to be statistically significant in our data. Additionally, we did not observe a statistically significant association between food insecurity and sleep onset latency. We hypothesize that, while sleep duration, sleep quality, and sleep onset latency are aspects of sleep health, they do not necessarily represent the same construct (Kohyama, 2021). Sleep duration and sleep onset latency may be reflective of physical aspects of one’s environment, and they may be more readily affected by environmental factors (e.g., light pollution and loud noises); sleep quality, on the other hand, may be reflective of biological mechanisms, and it may be more readily affected by biological factors (e.g., stress and nutritional intake) (Bin, 2016). Indeed, several studies have shown that sleep duration, sleep quality, and sleep onset latency are not perfectly correlated; some individuals can get adequate amounts of sleep, yet experience poor-quality sleep, while other individuals can get minimal amounts of sleep and feel rested (Seow et al., 2020). There may be specific pathways that connect food insecurity and sleep quality, including: the stress pathway, in that food insecurity can cause stress and psychological distress, which can lead to worse sleep health (Bermúdez-Millán et al., 2016; Ding et al., 2015); and nutritional pathway, in that those who experience food insecurity have to eat convenience food and ultra processed foods that are high in sugar and fats and low in nutrients, which can lead to poor sleep quality (Odoms-Young et al., 2024). Indeed, food-insecure individuals tend to have unhealthier diets characterized by lower intakes of plant-based foods, which are linked to worse sleep quality (St-Onge et al., 2016). Additionally, food insecurity is associated with a higher body mass index (Carvajal-Aldaz et al., 2022), which in turn is associated with sleep-disordered breathing symptoms that can worsen sleep quality (Leinum et al., 2009). These pathways may be irrelevant for sleep duration and sleep onset latency. Additional research can help elucidate the mechanisms that explain the relationship between food insecurity and sleep health, including the lack of association found with sleep duration and sleep onset latency.

The association between food insecurity and sleep health may partially explain why transgender women of color have disproportionately high rates of adverse health outcomes. Transgender women of color are vulnerable to economic deprivation that results in worse socioeconomic outcomes (Eastwood et al., 2021); transgender women of color, therefore, are more likely to experience food insecurity (Zubizarreta et al., 2024). In this study, we found that food insecurity is associated with adverse sleep health outcomes. Sleep, in turn, is associated with worse physical and mental health (Colten & Altevogt, 2006). Therefore, food insecurity and sleep health may partially explain some of the health inequalities that transgender women of color face (Seelman et al., 2017).

Food is foundational for good health– no one should be experiencing food insecurity. The fact that so many transgender women of color are experiencing food insecurity and potentially hunger needs to be addressed immediately. Given the results of our study as well as the abundance of research showing the health risks of food insecurity, we advocate for a structural approach to addressing food insecurity (Murray et al., 2023). Examples of structural approaches include protecting and bolstering the Supplemental Nutrition Assistance Program, also known as SNAP. This program offers financial services to qualifying participants to access food and is effective in mitigating food insecurity and hunger (Lee & Singh, 2022). However, the current federal administration has proposed to make cuts to federal funding for SNAP and make eligibility stricter, which could lead to fewer people receiving benefits and more people experiencing food insecurity (Rosenbaum et al., 2026). Continuing and strengthening SNAP is therefore paramount to prevent food insecurity and improve health outcomes among vulnerable populations, including transgender women of color. For mental health care providers, referring patients to food programs or social workers can also be effective for reducing food insecurity and improving mental health outcomes (De Marchis et al., 2019). Other evidence-based interventions, such as community food pantries and food delivery programs, can also help mitigate food insecurity (Burns et al., 2010; Smith et al., 2025).

We also believe that addressing systems of oppression, including racism, transphobia, and sexism, is necessary to get at the root cause of food insecurity and health inequalities (Baciu et al., 2017). Addressing residential segregation and disproportionate criminalization of transgender women of color can reduce food insecurity and lead to health equity (Bower et al., 2014; Odoms-Young & Bruce, 2018; Robles et al., 2024). Such approaches necessarily move the focus away from the individual to structural factors. We believe that an explicit reframing of this issue is necessary, as mainstream narratives around those requiring food assistance often “blame the victim.” Not only are those experiencing food insecurity suffering from hunger and its health consequences, but they are also often stigmatized and made to feel shame (Pineau et al., 2021; Russomanno et al., 2019; Swales et al., 2020).

### Limitations

We acknowledge that this study has several limitations. First, unmeasured confounding, as in all observational studies, may bias these results. While we controlled for age, education, income, US-born nativity, sex work history, and incarceration history, there may be potential unmeasured confounding that could be biasing our results. For example, we did not measure and adjust for chronic comorbidities, which may be common causes of food insecurity and of sleep health outcomes (Dikeos & Georgantopoulos, 2011). Second, we assessed self-reported food insecurity and sleep. Our measure of food insecurity may be crude, as it came from a single-item question. While the Hunger Vital Sign tool asks about whether or not someone experienced running out of food, we did not; we also did not measure duration of food insecurity, or severity of food insecurity (Nicholson et al., 2025). Additionally, we did not administer the entirety of the PSQI when measuring sleep health; this prevents us from using the validated instrument to its full potential. However, we note that other researchers have examined specific aspects of sleep health in isolation (He et al., 2025; Keramat et al., 2023); understanding sleep duration, sleep quality, and sleep onset latency separately can also be beneficial, as each may reflect different aspects of sleep health and may have its own mechanisms (Kohyama, 2021; Kripke et al., 2002). We further acknowledge that the PSQI does not allow us to assess specific sleep disorders; we recommend that future investigators use tools such as electroencephalography or wearable technologies to obtain objective measures of sleep disorders (Norris et al., 2023). Third, our sample may be vulnerable to selection bias due to the convenience sampling methodology, and the results may not be generalizable. The experiences of individuals in this sample may not be generalizable to all transgender women of color in New York City or the United States or more broadly across the globe. Fourth, our longitudinal analyses may be biased due to loss to follow-up. We checked if observed covariates changed over time and found that most variables stayed constant over time (Supplemental Table 1); however, there may be unobserved factors that were affected by loss to follow-up that may introduce bias in our longitudinal analysis. Finally, loss to follow-up results in a smaller sample size over time, meaning that we lost some statistical power in the 12-month analysis.

### Clinical significance

The high prevalence of food insecurity among transgender women of color needs to be considered as a public health emergency. Addressing food insecurity is paramount to reducing health burdens among transgender women of color—a population that faces intersecting structural vulnerabilities. Our study provides longitudinal evidence that food insecurity is associated with poorer sleep quality over time. We hypothesize that this relationship may be driven by stress pathways and nutritional deficiencies, both of which are shaped by broader social and economic inequities. These findings underscore the need for structural interventions, such as protecting and bolstering food benefit programs like SNAP and addressing residential segregation and disparities in criminal legal system involvement, that enhance food security. Mental health care providers should be cognizant of the importance of food insecurity and sleep health for patients who identify as transgender women of color; care providers can refer patients to food programs or social workers. Advancing such interventions is essential to mitigating the cumulative health burden faced by transgender women of color and promoting equity in health outcomes.

## Supplementary Material

Supp 1

Supplemental data for this article can be accessed online at https://doi.org/10.1080/30678935.2026.2713222.

## Figures and Tables

**Table 1.** Baseline characteristics of the study population, the TURNNT cohort study.

	% (*n* = 314)
Age	
18 to 24	10.2%
25 to 34	29.6%
35 to 44	37.9%
45 to 55	21.0%
Missing	1.3%
Education	
Less than High School	33.4%
High School	28.3%
More than High School	38.2%
Income	
No Income	16.2%
$1 to $9,999	39.2%
$10,000 to $29,999	22.0%
$30,000 or more	11.8%
Missing	10.8%
US-Born	
Yes	58.3%
No	41.1%
Missing	0.6%
Sex Work History	
Yes	75.2%
No	23.9%
Missing	1.0%
Incarceration History	
Yes	27.7%
No	71.3%
Missing	1.0%
Food Insecurity	
Yes	59.9%
No	39.2%
Missing	1.0%
Sleep Duration	
Sufficient Sleep (≥ 7 h)	45.5%
Short Sleep (< 7 h)	54.5%
Sleep Quality	
Very Good	18.2%
Fairly Good	45.9%
Fairly Bad	24.5%
Very Bad	10.5%
Missing	1.0%
Long Sleep Onset Latency^a^	
No	74.3%
Yes	25.2%
Missing	0.5%

The table shows the distribution of the sociodemographic characteristics, as well as food insecurity and sleep health outcomes, of the TURNNT cohort study.

a Long sleep onset latency was first measured at Wave 2, where *n* = 222.

**Table 2.** Correlates of food insecurity.

	Food secure % (*n* = 123)	Food insecure % (*n* = 188)	Prevalence ratio (95% CI)
Age			
18 to 24	9.8%	10.1%	Reference
25 to 34	27.6%	30.3%	1.02 (0.74, 1.41)
35 to 44	35.8%	39.9%	1.03 (0.75, 1.40)
45 to 55	24.4%	19.1%	0.89 (0.62, 1.27)
Education			
More than High School	44.7%	33.5%	Reference
High School	28.5%	28.2%	1.13 (0.89, 1.43)
Less than High School	26.8%	38.3%	**1.28 (1.04, 1.59)**
Income			
$30,000 or more	19.5%	6.9%	Reference
$10,000 to $29,999	18.7%	23.4%	**1.87 (1.17, 2.99)**
$1 to $9,999	39.8%	39.4%	**1.71 (1.08, 2.71)**
No Income	10.6%	20.2%	**2.12 (1.33, 3.38)**
Missing	11.4%	10.1%	1.64 (0.97, 2.77)
US-Born			
Yes	39.8%	42.6%	Reference
No	59.3%	56.9%	0.96 (0.80, 1.15)
Sex Work History			
No	26.0%	21.8%	Reference
Yes	73.2%	77.1%	1.10 (0.88, 1.38)
Incarceration History			
No	73.2%	69.7%	Reference
Yes	26.8%	28.7%	1.05 (0.86, 1.28)

We calculated the prevalence ratios to determine whether sociodemographic characteristics (age, education, income, US-nativity, sex work history, and incarceration history) were correlates of food insecurity. Bold cells indicate that the association is statistically significant at the level of significance of 0.05.

**Table 3.** Longitudinal associations between food insecurity and sleep health using doubly-ro-bust estimators.

Longitudinal association			Adjusted risk ratio [95% confidence interval]	Analytical sample size
Baseline Food Insecurity	↔	6 Month Short Sleep	0.93 (0.72, 1.19)	210
Baseline Food Insecurity	↔	12 Month Short Sleep	1.20 (0.93, 1.54)	187
Baseline Food Insecurity	↔	6 Month Poor Sleep	**2.03 (1.26, 3.29)**	206
Baseline Food Insecurity	↔	12 Month Poor Sleep	1.36 (0.84, 2.20)	185
Baseline Food Insecurity	↔	6 Month Long Sleep Onset Latency	1.17 (0.73, 1.87)	210
Baseline Food Insecurity	↔	12 Month Long Sleep Onset Latency	1.56 (0.94, 2.58)	188

Using doubly-robust estimators, we estimated the longitudinal association between food insecurity and sleep health outcomes (short sleep, poor sleep quality, and long sleep onset latency), adjusting for age, education, income, US-nativity, sex work history, and incarceration history. Bold cells indicate that the association is statistically significant at the level of significance of 0.05.

## Data Availability

The data that support the findings of this study are available from the corresponding author, upon reasonable request.
