# Supplementary material for: The association between food insecurity and poor sleep quality among transgender women of color: The TURNNT cohort study
Source: J LGBTQ+ Ment Health. Author manuscript; Available in PMC 2026 Sep 30. (PMC13621917; doi:10.1080/30678935.2026.2713222)
Supplement: Supplementary file 1 [file NIHMS2207186-supplement-Supp_1.docx]

**Supplemental Table 1: Characteristics of the Study Population over Time**

|  | Baseline | 6-Month Follow-Up | 12-month Follow-Up | Chi-Squared  Test |
| --- | --- | --- | --- | --- |
|  | % (n=314) | % (n=218) | % (n=193) | p-value |
| Baseline Age |  |  |  | 0.86 |
| 18 to 24 | 10.2% | 10.1% | 7.3% |  |
| 25 to 34 | 29.6% | 28.9% | 28.5 |  |
| 35 to 44 | 37.9% | 35.8% | 37.3% |  |
| 45 to 55 | 21.0% | 23.9% | 25.4% |  |
| Missing | 1.3% | 1.4% | 1.6% |  |
| Baseline Education |  |  |  | 0.99 |
| Less than High School | 33.4% | 32.6% | 32.6% |  |
| High School | 28.3% | 29.4% | 28.0% |  |
| More than High School | 38.2% | 38.1% | 39.4% |  |
| Baseline Income |  |  |  | 0.99 |
| No Income | 16.2% | 16.5% | 14.0% |  |
| $1 to $9,999 | 39.2% | 38.1% | 38.9% |  |
| $10,000 to $29,999 | 22.0% | 21.1% | 22.8% |  |
| $30,000 or more | 11.8% | 12.4% | 13.5% |  |
| Missing | 10.8% | 11.9% | 10.9% |  |
| Baseline US-Born Nativity |  |  |  | 0.88 |
| Yes | 58.3% | 60.6% | 59.1% |  |
| No | 41.1% | 39.0% | 40.4% |  |
| Missing | 0.6% | 0.5% | 0.5% |  |
| Baseline Sex Work History |  |  |  | 0.94 |
| Yes | 75.2% | 74.8% | 76.2% |  |
| No | 23.9% | 24.8% | 23.3% |  |
| Missing | 1.0% | 0.5% | 0.5% |  |
| Baseline Incarceration History |  |  |  | 0.99 |
| Yes | 27.7% | 27.1% | 27.5% |  |
| No | 71.3% | 71.6% | 72.0% |  |
| Missing | 1.0% | 1.4% | 0.5% |  |
| Food Insecurity |  |  |  | 0.75 |
| Yes | 59.9% | 57.3% | 59.6% |  |
| No | 39.2% | 42.2% | 38.3% |  |
| Missing | 1.0% | 0.5% | 2.1% |  |
| Sleep Duration |  |  |  | 0.71 |
| Sufficient Sleep (≥ 7 Hours) | 45.5% | 46.8% | 42.5% |  |
| Short Sleep (< 7 Hours) | 54.5% | 52.8% | 56.5% |  |
| Missing | 0.0% | 0.5% | 1.0% |  |
| Sleep Quality |  |  |  | 0.26 |
| Very Good | 18.2% | 18.3% | 14.0% |  |
| Fairly Good | 45.9% | 50.0% | 55.4% |  |
| Fairly Bad | 24.5% | 17.4% | 19.7% |  |
| Very Bad | 10.5% | 11.9% | 8.8% |  |
| Missing | 1.0% | 0.5% | 0.5% |  |
| Long Sleep Onset Latency |  |  |  | 0.60 |
| No | - | 74.3% | 71.5% |  |
| Yes | - | 25.2% | 28.0% |  |
| Missing | - | 0.5% | 0.5% |  |

**Supplemental Table 2: Unadjusted Longitudinal Associations Between Food Insecurity and Sleep Health.** We calculated risk ratios to estimate the longitudinal association between food insecurity and sleep health outcomes (sleep duration, poor sleep quality, and long sleep onset latency).

| **Longitudinal Association** | | | **Risk Ratio**  **[95% Confidence Interval]** | **Analytical**  **Sample Size** |
| --- | --- | --- | --- | --- |
| Baseline  Food Insecurity | ↔ | 6 Month  Short Sleep | 0.97  (0.75, 1.25) | 215 |
| Baseline  Food Insecurity | ↔ | 12 Month  Short Sleep | 1.27  (0.96, 1.68) | 190 |
| Baseline  Food Insecurity | ↔ | 6 Month  Poor Sleep | **1.94**  **(1.18, 3.19)** | 211 |
| Baseline  Food Insecurity | ↔ | 12 Month  Poor Sleep | 1.41  (0.86, 2.33) | 188 |
| Baseline  Food Insecurity | ↔ | 6 Month  Long Sleep  Onset Latency | 1.12  (0.69, 1.80) | 215 |
| Baseline  Food Insecurity | ↔ | 12 Month  Long Sleep  Onset Latency | 1.37  (0.83, 2.28) | 191 |
